# Priorities and preferences for care of people with multiple chronic conditions

**DOI:** 10.1111/hex.13262

**Published:** 2021-05-03

**Authors:** Mieke Rijken, René Stüssgen, Chantal Leemrijse, Mieke J. L. Bogerd, Joke C. Korevaar

**Affiliations:** ^1^ Nivel (Netherlands Institute for Health Services Research) Utrecht The Netherlands; ^2^ Department of Health and Social Management University of Eastern Finland Kuopio Finland; ^3^ Department of General Practice & Elderly Care Medicine Amsterdam Public Health research institute Amsterdam UMC, location VUmc Amsterdam The Netherlands; ^4^ Zorginstituut Nederland (National Health Care Institute) Diemen The Netherlands

**Keywords:** delivery of health care, health priorities, integrated, multiple chronic conditions, patient preference, primary health care, qualitative research, survey

## Abstract

**Background:**

To guide the development of high‐quality care for people with multiple chronic conditions, partners of the European Joint Action CHRODIS developed the Integrated Multimorbidity Care Model. To assess its suitability for improving care for people with multimorbidity in the Netherlands, the model was piloted in a primary care setting with both patients and care providers.

**Aim:**

This paper reports on the patient perspective, and aims to explore the priorities, underlying values and preferences for care of people with multimorbidity.

**Participants and methods:**

Twenty persons with multimorbidity (selected from general practice registries) participated in a focus group or telephone interview. Subsequently, a questionnaire was completed by 863 persons with multimorbidity registered with 14 general practices. Qualitative data were thematically analysed and quantitative data by means of descriptive statistics.

**Results:**

Frequently prioritized elements of care were the use of shared electronic health records, regular comprehensive assessments, self‐management support and shared decision making, and care coordination. Preferences for how these elements should be specifically addressed differed according to individual values (eg weighing safety against privacy) and needs (eg ways of coping with multimorbidity).

**Conclusion:**

The JA‐CHRODIS Integrated Multimorbidity Care Model reflects the priorities and preferences for care of people with multimorbidity in the Netherlands, which supports its relevance to guide the development of person‐centred integrated care for people with multiple chronic conditions in the Netherlands.

**Patient contribution:**

European patient experts contributed to the development and applicability assessment of the JA‐CHRODIS Integrated Multimorbidity Care Model; Dutch patients participated in focus groups, interviews and a survey.

## 1 INTRODUCTION

After the wake‐up call by the World Health Organization in 2002 that chronic conditions would be the biggest challenge for health systems worldwide in the new millennium,^1^ chronic care today requires reform and innovation once again, now because of the growing populations with *multiple* chronic conditions. In some countries, the number of people with two or more chronic conditions already exceeds the number of people with one chronic condition. ^2^, ^3^, ^4^, ^5^ Awareness has raised that the multifaceted nature of multimorbidity calls for alignment of services that go beyond (single) disease management programmes and single‐disease chains of care,^6^, ^7^, ^8^ necessitating intersectoral collaboration as well, particularly with social care and community services. Moreover, people with multimorbidity need person‐centred care, which requires care professionals to take up new roles and develop new skills.^9^, ^10^, ^11^

### 1.1 Integrated Multimorbidity Care Model

Similar to the previous situation with the management of (single) chronic conditions, new care models are being developed to guide the development and implementation of person‐centred integrated care for people with multiple chronic conditions. One of these models is the Integrated Multimorbidity Care Model (IMCM), which was developed by the European Joint Action CHRODIS based on previous integrated care models,^12^, ^13^, ^14^ scientific evidence^15^ and consultation of international experts. This resulted in agreement on 16 components of high‐quality multimorbidity care, structured under five main components (Box 1).^16^ Pilots in Italy, Lithuania and Spain that were conducted as part of the European Joint Action CHRODIS+^17^ show the potential of the model to improve care for people with multimorbidity in various settings and in countries with different health systems.^18^

BOX 1

Components of the integrated multimorbidity care model (see Palmer et al., 2018 for detailed description of components)

**Table:** 

**Delivery system design**		**Clinical information systems**	
1	Regular comprehensive assessment	11	Electronic patients records and computerized clinical charts
2	Multidisciplinary team	12	Exchange of patient information
3	Individualized care plans	13	Uniform coding of patients’ health problems
4	Appointment of care coordinator or case manager	14	Patient platforms allowing patients to exchange information with care providers
**Decision support**		**Community resources**	
5	Implementation of evidence‐based medicine	15	Access to community resources
6	Team training	16	Involvement of social network
7	Development of a consultation system		
**Self‐management support**			
8	Training care providers to tailor self‐management support based on patient preferences and competencies		
9	Providing options for patients and families to improve self‐management		
10	Involvement of patients in decision making		

### 1.2 Patient voice

Although patient representatives participated in the expert consultation, the patient voice in the development of the IMCM has been limited until now. Some previous studies reported priorities and preferences for care of people with multimorbidity. A survey in the United States among primary care patients to evaluate the implementation of a new team‐based care model demonstrated that, although all patients considered continuity of care very important, people with multimorbidity attached even greater value to it.^19^ A qualitative study in Canada and New Zealand showed that older participants with multimorbidity attached high value to good patient‐provider communication (eg feeling heard; getting enough time; attention paid to their comprehensive needs and priorities), having a trusted care provider, knowledge to self‐manage health and care, and smooth access to health and social services.^20^ Another qualitative study, in the United States, confirmed that older people with multimorbidity value being heard and a person‐centred communication and a single care provider coordinating their care and helping them prioritize competing demands.^21^ Although these results support the relevance of certain components of the IMCM, empirical evidence from other, also European, countries is needed, since values and care preferences of people with multimorbidity may be context‐ and culture‐sensitive.^22^

To contribute to the empirical evidence of the model as a basis for multimorbidity care development and to further explore its relevance from the patient perspective, we conducted a mixed‐methods study guided by the following research questions:

1. Which components of person‐centred integrated care as described by the IMCM are prioritized by community‐dwelling persons with multimorbidity in the Netherlands?
2. Which preferences do community‐dwelling people with multimorbidity have regarding the practical implementation of the prioritized components in care provided by Dutch general practices and their local partners?

These questions are also relevant considering that implementing person‐centred integrated care in real‐life care settings will often require a stepwise approach over a number of years. Knowing patients’ priorities may be helpful to set goals and select a first set of interventions to start with.

## 2 METHODS

A mixed‐methods study was conducted to make optimal use of the benefits of both qualitative and quantitative research methods. To assess patients’ priorities for multimorbidity care (research question 1), we considered a qualitative study design most appropriate as it allowed us to collect and analyse rich unstructured data, also because we aimed to identify the values underlying participants' priorities. Therefore, we organized face‐to‐face focus groups and telephone interviews with people with multimorbidity. To assess patients' preferences for the concrete implementation of the prioritized components of care (research question 2), we conducted a survey among a larger group of persons with multimorbidity using a structured questionnaire. In this way, we were able to examine patients’ preferences for a number of care interventions that could actually be implemented by Dutch general practices and their local partners.

### 2.1 Focus group/interview study

#### 2.1.1 Participants

Participants were recruited from the National Panel of people with Chronic illness or Disability (NPCD). ^23^, ^24^ In this panel study, about 1500 to 2000 adults with chronic conditions, randomly selected from general practices throughout the Netherlands, participate each year in surveys to monitor developments in chronic care and social participation. Occasionally, participants are invited for focus groups or interviews to collect more in‐depth information.

In February 2018, we sent an invitation letter explaining the purpose and methods of the study to 314 panel members (aged 18+) who were randomly selected from the total group of 631 panel members eligible for this study, that is diagnosed with at least two chronic diseases and not having participated in a focus group or (telephone) interview over the last year. Though we preferred focus groups above telephone interviews, expecting that the interactive nature of focus groups would encourage participants to also reflect on other views than their own, we also offered the option for a telephone interview to reduce barriers for participation (particularly because of the possibly poor functional health status of the invited persons) to be as inclusive as possible.

Eight persons indicated their willingness to participate in a face‐to‐face focus group and 37 in a telephone interview (total 45; 14%). As two persons were not able to participate in a focus group on the dates we could offer, six actually participated in a focus group. One group consisted of two participants and the other of four. Of the 37 persons who expressed their interest in a telephone interview, we randomly selected persons, and conducted and analysed interviews until we felt that data saturation had been reached. This was after 14 interviews (in addition to the focus groups). In addition to the information letter they received, we further explained the study verbally before the start of the focus group or interview and answered questions; all participants signed informed consent.

#### 2.1.2 Data collection

Each focus group was moderated by two researchers; the telephone interviews were conducted by three interviewers. To guide the focus groups, we developed ten statements (‘For me it is important that …’; see Table 1), based on the five main components of the IMCM.^16^ Each statement was printed on an A3 poster and hang (in a random order) in the room where the focus groups were held. After an introduction explaining the goal of the focus group, the ground rules and getting to know each other, all participants received three post‐it notes and were instructed to stick them on the posters that reflected best what they felt most important in caring for their multiple chronic conditions. After all participants had selected three statements, the moderators started with the statements that had been chosen most frequently and invited the participants to explain why they had (not) chosen them. Participants were encouraged to join the conversation and add to or comment on each other's explanations or reasons. In a similar way, participants discussed the least frequently chosen statements.

**TABLE 1:** Statements related to IMCM components; frequency of statement reported among top 3 priorities of participants (N = 20)

IMCM component	*For me it is important that …*	n
**Delivery system design**		
Regular comprehensive assessment	1. … one of my care providers assesses my whole situation regularly (for instance, every six months); and not only my medical situation, but also explores how I feel, what my daily life looks like and whether I can manage at home and in daily life.	12
Individual care plan	2. … one of my care providers develops a personal care plan with me, based on my needs and preferences; which not only focuses on the management of my chronic conditions, but also addresses my broader needs and way of living, including my social contacts and living environment	3
Care coordinator	3. … one of my care providers is the link between me, my important others/carers and all other care providers of which I receive care or support; thus, one person who will liaise and manages all information about my care	6
**Decision support**		
Consultation system	4. … my care providers consult experts (for instance, medical specialists, psychologists, etc), if they lack knowledge or experience to decide about the best care or treatment for me	2
**Self‐management support**		
Provide options for self‐management	5. … my care providers support me in retaining control and taking care of myself	8
Shared decision making	6. … my care providers encourage me to actively consider my options for care and treatment and engage in shared decision‐making about my care	5
**Information systems and technology**		
Patient‐operated technology	7. … my care provider considers eHealth technology as an option for me; for instance whether I could use an app or website to send health monitoring information to the health center or hospital, so that less face‐to‐face visits will be needed	3
Shared electronic health records	8. … my care providers register my health and care data in one electronic file, so that they have quick access to all relevant information	15
**Social and community resources**		
Assessment of support needs	9. … my care provider asks about my needs for support, for example my need for domestic help, transport facilities, exercise programmes or peer support	4
Access to community and social resources	10. … my care provider informs me about community resources for support and activities in my neighbourhood, and helps me getting in touch	2

For the telephone interviews, participants received a set of ten cards (each containing one of the ten statements; randomly ordered) by post some days before the interview with a letter asking them to read the statements beforehand and select three cards that best reflected what they valued most in caring for their multiple chronic conditions. During the interview, the interviewer invited the interviewee to explain his/her choice for the statements. Dependent on how much time this had taken, interviewees were also encouraged to comment on the statements they had not prioritized.

The focus groups and telephone interviews were audiotaped, with permission of the participants. The audiotapes were transcribed verbatim by an independent transcription service; all identifying information was removed from the transcripts.

#### 2.1.3 Data analysis

Descriptive statistics of participants' characteristics and selected statements were calculated. The transcripts were thematically analysed^25^; the components of the IMCM were included as predefined codes for all transcripts (transcripts of the focus groups and telephone interviews were analysed together), but new codes were added for phrases that were not covered by the predefined codes. Only phrases in the transcripts that related to the concept of quality of care were coded. Initial coding of all transcripts was done by one researcher and checked by a second researcher. Differences in interpretation of (un‐)coded phrases were discussed with a third researcher, if necessary to decide on initial coding. Subsequently, higher‐order themes were identified based on these codes. As we were interested in participants' values behind their prioritization of certain aspects of multimorbidity care, which are usually not explicitly expressed, we examined codes including participants’ explanations of why they prioritized certain aspects of care. The preliminary identified themes were reviewed by re‐reading all relevant phrases of the transcripts and checked for alternative explanations. The final themes included both semantic and latent themes.

### 2.2 Survey

#### 2.2.1 Participants

Participants were selected from the clinical information systems of fourteen general practices that participated in a quality improvement project based on the IMCM. Inclusion criteria were (a) aged 18 and over, and (b) at least three conditions from a list of 56^1^ chronic conditions. This list had been developed in multiple brainstorm sessions with Dutch general practitioners (GPs) participating in a quality improvement project^26^ who selected conditions they considered as ‘in need of chronic primary care’ and checked with another internationally used list of chronic conditions.^27^ This resulted in an initial sample of 2517 selected persons, of which GPs excluded persons whom they felt incapable of participating in the survey because of severe mental or physical health problems. As not all GPs kept a registry of excluded persons, the exact total number of excluded persons is unknown, but estimated around 300, implying a final sample of about 2200 persons with three or more chronic diseases.

#### 2.2.2 Data collection

A paper questionnaire together with an information letter explaining the purpose and methods of the study was sent by the general practices to the included persons. No reminders were sent. Persons returned their completed questionnaire directly and anonymously to the research institute, without interference of the general practices. Data collection and processing were in accordance with the Dutch Code of Conduct for the Use of Data in Health Research (https://www.federa.org/codes‐conduct). According to the Central Committee on Research involving Human Subjects (CCMO), this type of study does not require approval from an ethics committee in the Netherlands.

The questionnaire contained seven questions (see Table 2) developed by the authors, to assess the preferences of the respondents regarding those components of the IMCM that had been prioritized most frequently by the participants in the focus groups and telephone interviews. Respondents were allowed to choose more than one answering option per question. Furthermore, they could explain their answers in an open text box. Additional data collected by the questionnaire were age, gender and health‐related quality of life as assessed with the first item of the RAND‐36^28^: In general, how would you say your health is? Response options are as follows: excellent, very good, good, moderate and poor.

**TABLE 2:** Preferences of people with three or more chronic conditions for receiving care for their chronic conditions

		N	n	%
IMCM component: Shared electronic health records				
1.	Doctors that have medical data of me …			
‐	are not allowed to share my data with each other, unless I give permission in a certain situation	832	70	8.4
‐	can share my data with each other, but it must be limited to what is really necessary	832	223	26.8
‐	are allowed to share my data with each other, but not with health‐care providers who are not physicians (for example, the physical therapist)	831	75	9.0
‐	may share my data with all health‐care providers involved in caring for my conditions	831	338	40.7
‐	can also share my data with organizations or persons who have to assess my situation for support from the municipality (for example, for domestic help)	831	162	19.5
‐	other	831	15	1.8
IMCM component: regular comprehensive assessments				
2.	I want a comprehensive assessment of my health and functioning …			
‐	when I find it necessary	851	309	36.3
‐	when my GP finds it necessary	851	290	34.1
‐	when my medical specialist finds it necessary	851	103	12.1
‐	every year anyway	851	200	23.5
‐	Other	851	42	4.9
3.	A comprehensive assessment of my health and functioning …			
‐	must be limited to my physical health	813	217	26.7
‐	should also be about my memory or mood, in addition to my physical health	813	160	19.7
‐	should be about my entire situation, so not only about my medical situation and how I feel, but also how it goes at home and in my daily life	813	442	54.4
‐	Other	813	21	2.6
IMCM component: self‐management support, including shared decision making				
4.	I prefer advice and support to manage my conditions well …			
‐	to receive from the GP	826	581	70.3
‐	to receive from the nurse practitioner / practice nurse	826	149	18.0
‐	to receive from the medical specialist	826	232	28.1
‐	to receive from the nurse in the hospital	826	36	4.4
‐	to receive from the community nurse	825	23	2.8
‐	to receive from someone who is a patient himself	826	15	1.8
‐	to receive from someone else, namely …	826	42	5.1
‐	not to receive	823	20	2.4
5.	To learn how to best self‐manage my conditions …			
‐	I would like to have conversations with the GP or nurse practitioner / practice nurse	806	511	63.4
‐	I (also) want to participate in group discussions with other patients, led by my GP or practice nurse	805	57	7.1
‐	I (also) want to participate in a course led by another health‐care provider, for example a psychologist	806	49	6.1
‐	I (also) want to participate in a course led by an experienced patient	806	26	3.2
‐	I (also) want to follow a course via the internet	806	18	2.2
‐	I don't want to have extra conversations or follow a course	806	178	22.1
‐	Other	806	69	8.6
6.	I prefer decisions about the care or treatment I receive			
‐	to make as much as possible myself (if necessary together with my loved ones)	851	316	37.1
‐	to make as much as possible together with the GP	851	421	49.5
‐	to make as much as possible together with the specialist	851	153	18.0
‐	to leave them to the GP	851	38	4.5
‐	to leave them to the medical specialist	851	24	2.8
‐	Other	851	35	4.1
IMCM component: care coordination				
7.	I prefer organizing the care or support I need …			
‐	to do it myself (if necessary together with my loved ones)	849	509	60.0
‐	to leave it to the GP or nurse practitioner / practice nurse	849	245	28.9
‐	to leave it to the medical specialist or nurse in the hospital	849	71	8.4
‐	to leave it to the community nurse	849	15	1.8
‐	other	849	72	8.5

#### 2.2.3 Data analysis

Univariate statistics were computed to describe the demographic characteristics of the participants and to assess their preferences for care.

## 3 RESULTS PART I: FOCUS GROUP/INTERVIEW STUDY

### 3.1 Participants

The group consisted of thirteen women and seven men. The mean age was 68.2 (SD: 12.3; range: 40‐89). Many participants reported three or more chronic conditions, both somatic (eg diabetes, ischaemic heart disease, cancer, arthritis, asthma, COPD, multiple sclerosis, thyroid disorder) and mental conditions (anxiety disorder, ADHD, depression). Twelve persons (60%) evaluated their general health as moderate; five as good (25%); and the other three as excellent (5%), very good (5%) or poor (5%).

### 3.2 Components of multimorbidity care most valued by the participants

Table 1 shows the ten statements related to the IMCM components and how frequently each of them had been prioritized by the participants (among their top 3 priorities). It shows that participants attached high value to having one health record shared by all care providers involved in their care (prioritized by 15 of the 20 participants), regular comprehensive assessments (prioritized by 12 participants) and receiving support from their care providers to self‐manage their chronic conditions (prioritized by 8 participants). Care coordination was prioritized by six participants and shared decision making by five.

Below, we describe the results of the qualitative analysis referring to the most frequently prioritized components.

#### 3.2.1 Shared electronic health records

Three quarters of the participants wanted to have all their health data in one electronic record shared by multiple care providers. Participants were less unanimous about which parts of their data to share with whom. Some participants would favour to share as much information as possible with as many of their care providers: ‘*Just pressing a single button and they have all your data. Isn't* *that wonderful?’* When asked which care providers should get access to their medical information, these people not only mentioned their GP, pharmacist and other medical specialists, but also their physiotherapist, dietician, dentist and many more. Some felt that social care and community services should also have direct access to their health record(s). Others mentioned conditions and restrictions for sharing their data. These people wished to be asked for permission each time their data were shared with or transferred to another care provider; they mentioned restrictions regarding the type or amount of data to be shared or regarding the type of care providers data could be shared with.

The different attitudes of participants towards sharing their health data seem to be related to how they value and balance continuity and safety of care against privacy. Participants who strongly favoured unlimited exchange of their data emphasized that this would improve the quality and efficiency of their consultations with care providers and decrease the risk of inappropriate or even harmful care interventions because of care providers missing essential information. Some of them also mentioned they did not want to be responsible for accurate information transfer between care providers; others just did not like to tell their story over and over again. There were also participants who did not favour unlimited exchange of their health data, however. These people seemed to be more inclined to protect their privacy. One participant stated that she did not want every care provider to know about her mental health problem, as she felt this would influence their perception of her physical problems: ‘*On the one hand, one electronic patient record would be very useful, as it is then known to all of them which allergies I have, and why and how and what. But on the other hand, I don't want every doctor to know that I have a panic disorder. Not that I'm ashamed of it, but when I come up with something physical, they may tend to say: Oh yes, yes … no, but that's a panic disorder’*.

Some participants felt that a personal health page at a secured IT platform or an electronic health passport by which they could access their data and decide themselves which parts to share with their care providers would be a good solution. Several participants reported to have access to their health data via a patient portal, but that the GP and hospital portals were not linked.

#### 3.2.2 Regular comprehensive assessments

Most people who gave high priority to regular comprehensive assessments felt it important to discuss their whole situation, not only their medical condition, with a trusted care provider now and then, as they felt that ‘*everything is connected’*. One participant also mentioned to value regular evaluations of her self‐management and coping: ‘*I think it is very important that a doctor regularly checks what I do right or wrong, or whatever. That he just assesses me. And also mentally, because how do you handle everything?’* One participant who did not give priority to regular comprehensive assessments explained she felt no need: ‘*That is less applicable to me. I am actually in good health’*.

Prioritizing regular comprehensive assessments seems to be related to two outcome expectations: first, a belief that such assessments could provide points of action for prevention, either for behavioural responses of people with multimorbidity themselves or for appropriate treatment or care provided by professionals, and second, regular comprehensive assessments were prioritized because of a belief that they can provide assurance and trust in adequate management of conditions. Some participants held both beliefs; others seemed to expect either behavioural (preventive action) or more emotional (assurance) outcomes of regular comprehensive assessments.

The GP was mentioned most frequently as the care provider most suitable for this task. To explain their preference for the GP, participants pointed to the long‐term relationship they had with their GP, the good contact and the familiarity of the GP with their situation. One participant explained: ‘*Because then you have personal contact with the person who knows you, who also knows your background a bit and I think it is important to exchange ideas with such a person’*. Four participants preferred another care professional for the same reason. One participant mentioned a community nurse assessing her needs: ‘*And now she comes every six months. And she discusses with me what has deteriorated, and what I like. And whether I agree with the current treatments. And I really like that’*. This citation also illustrates the link that several participants spontaneously made between regular comprehensive assessments and individual care planning and monitoring.

#### 3.2.3 Self‐management support and shared decision making

Eight participants prioritized options for self‐management support, and five (also) prioritized shared decision making. As most participants did not make a clear distinction between these elements of care, they are described in one section.

Most participants who prioritized self‐management support from their care providers attached great value to being in control of their health and care. Most participants explained why they valued personal control rather than explaining whether and why they valued *being supported* to keep control. Several participants felt that keeping control over their health and care was essential to function as much as possible and for as long as possible independently, also including independent living. One participant felt he had to be in control over his health and care out of necessity: ‘*I prefer to know what has been done and what needs to be done, because we do run into things that go wrong. So, we need to keep an eye on these things ourselves’*. For this participant, the value of personal control was inversely related to his care experiences and trust in health care.

Several participants stated they felt it important that care providers encourage them to keep control and provide options for how they could self‐manage their health and care. However, only one person reported that this actually happened. All others reporting their experiences were less positive: ‘*I do not notice that my GP supports me’, ‘Yes, that is very important, but it does not happen as he is way too busy’* and ‘*No I am not supported in keeping control over my health, nor is it necessary yet. But I’m getting older, so I wonder: how long will this do?’*

Experiences with shared decision making were more positive, at least of those who attached high value to this care component. These participants considered shared decision making very important as they felt they take other aspects into account than their care provider(s) when considering the pros and cons of treatment options. For example, several participants mentioned that treatment burden and functioning were very decisive for them to start or stop a treatment, whereas their care providers seemed to focus more on clinical outcomes. ‘*They generally have a tendency to say: Well, if you look at those values, you better take an extra pill. And then I have to take two tablets for the sugar, for example, and I don't really want that. Because I always react so hard to medicines and stuff. Then she says: All right, then we'll go on like this’*. Other factors that were weighted differently in decision making between patients and care providers were related to patients’ psychological well‐being. One participant mentioned a clinical test as an example, which seemed unnecessary from a medical point of view, as its result would not make a difference for the care plan, but which he nevertheless wanted for reassurance.

#### 3.2.4 Care coordination

All participants considered coordination of care provided by different care providers very important, but not all felt a need for a professional taking the role of care coordinator; several participants mentioned their own role, or that of a family member, in coordinating their care. None of the participants explicitly expressed why they believed care coordination to be important. Based on the discussion in the two focus groups, continuity of care and efficiency seemed to be underlying values, which also came to the fore in conversations on data sharing via electronic health records.

Most participants were receiving care from more than one medical specialist and did not observe signs of communication between their specialists. A lack of communication was also felt between health‐care and social care providers. Moreover, some participants mentioned that care professionals communicated poorly with them and/or their family, which they felt undesirable considering their key role in managing their health and care.

Most participants pointed to the GP as the professional who could or should coordinate their care. Two participants believed it would be more efficient if other medical specialists communicate with each other directly, without the involvement of the GP. One participant reported positive experiences with an outpatient clinic providing all multidisciplinary care combined on one day. Some participants also mentioned observed barriers for care coordination, such as care providers lacking time, not being paid for coordinating tasks or not being recognized by other care providers as coordinator.

## 4 RESULTS PART II: SURVEY

### 4.1 Participants

A total of 863 patients with three or more chronic conditions returned the questionnaire (estimated response about 39%), though not all of them answered all questions. Among those who provided information about gender and age were 440 women (57%) and 326 men (43%). The mean age was 70.5 (SD: 11.6; range: 22‐96 years). Six per cent of the participants evaluated their general health as poor and 41% as moderate. A small majority (53%) were more positive about their health, with 45% evaluating their general health as good, 6% as very good and 2% as excellent.

### 4.2 Care preferences regarding prioritized IMCM components

Participants were asked to indicate their preferences for care regarding those IMCM components that had been prioritized most frequently by participants in the qualitative study. The results are presented in Table 2; below, we describe the most significant findings.

#### 4.2.1 Shared electronic health records

Forty per cent of all respondents reported that physicians should be able to share their medical information with all health‐care providers involved they had contact with. Another 20% indicated that their medical data could also be shared with organizations/persons responsible for the assessment of their needs for support provided by the municipality, such as domestic help. At the same time, more than a quarter of the respondents indicated that the exchange of data among care providers should be limited to what is really necessary.

#### 4.2.2 Regular comprehensive assessments

More than a third of the respondents indicated they want a comprehensive assessment of their health and functioning when they feel this is necessary. These people explained that they know their own bodies best and that they want to determine themselves when a comprehensive assessment is needed. A similar proportion mentioned that they (also) want such an assessment if their GP considers this necessary. Arguments of this group are that the GP knows what is going on, has the expertise and that they trust the GP’s judgement. Almost a quarter of all respondents preferred to have a comprehensive assessment of their health and functioning every year. These people explained they consider this important from a preventive point of view, that they want to be reassured or that they consider it necessary because of their age.

In terms of comprehensiveness, about half of all respondents preferred an assessment to cover their physical and mental health and their functioning at home and in daily life. As an explanation of their preference for a broad assessment, respondents indicated that they consider it important that 'the whole picture' is seen and that everything influences each other. On the other hand, a substantial group (27%) indicated a preference for an assessment limited to their physical health.

#### 4.2.3 Self‐management support and shared decision making

The vast majority of the respondents (70%) preferred to receive advice and support to self‐manage their health from their GP. More than a quarter (28%) also indicated they wished to get such advice and support from another medical specialist. There was less enthusiasm for self‐management support provided by a nurse working in general practice.

Most respondents (63%) preferred to receive self‐management support through bilateral conversations with their GP (or nurse practitioner/practice nurse). There was very little interest in other methods, such as group conversations or following a course. One in five indicated to not want extra conversations or a course to strengthen their self‐management at all. The main arguments of these participants were that they do not need it and ‘*do not see the added value’*. Someone else explained: ‘*I have already done everything’*. Respondents who chose the ‘other’ answering option most often explained they did not need support for self‐management.

Half of all respondents indicated a preference for decision making regarding treatments and care together with their GP. More than a third (37%) want to decide for themselves as much as possible, and about one in five (18%) would rather do this in consultation with another medical specialist. Only a small group indicated that they prefer to leave decisions to the GP or specialist.

#### 4.2.4 Care coordination

Most respondents indicated a preference for organizing the care and support they need themselves (60%). There was also a substantial proportion (29%) who prefer their GP (or nurse practitioner/practice nurse) to take the role of care coordinator. These people explained that they are in good contact with their GP, that it feels familiar, that the GP knows them well and that GP and practice nurse have the necessary expertise and short lines of communication. Respondents who chose the 'other' option (9%) predominantly indicated that they do not need any care or support; a few mentioned they had no idea who could organize their care or support.

## 5 DISCUSSION

This paper reports on the priorities and preferences for care of people with multimorbidity living in the Netherlands. Certain elements of care were frequently prioritized by interviewees and participants with multimorbidity: the use of shared electronic health records, regular comprehensive assessments, self‐management support and an active role of the patient in decision making, and care coordination. These elements relate to three of the five main components of the JA‐CHRODIS Integrated Multimorbidity Care Model; elements related to the other main components (decision support for care providers and integrating community services) were less frequently prioritized.

### 5.1 Discussion of main findings

Qualitative analysis revealed that multimorbid patients’ attitudes towards *shared electronic health records* depend on how much value they attach to either continuity of care and safety or their privacy. This was confirmed by the survey results, demonstrating substantial support for shared electronic health records and exchange of medical data among care providers, but also caution to which care providers should get access to which data.

*Regular comprehensive assessments* were felt important from a preventive point of view, but also as a way of being reassured about one's health status. These values may reflect different coping styles: proactive, problem‐focused coping versus reactive, emotion‐focused coping.^29^, ^30^ In the latter, regular assessments may be useful to reduce emotional discomfort, whereas in the former they may provide starting points for proactive (self‐)management. Both ways of coping could co‐exist among people with multimorbidity,^31^ but not necessarily. This may also explain why individual care plans, which are being used, amongst other things, to support patients’ self‐management,^32^ did not get an equally high priority as regular comprehensive assessments.

Participants in the qualitative study who gave high priority to *self‐management support* valued being in control of their health and care, but not all for the same reasons. Some pointed to the importance of self‐management to maintain independent functioning, whereas keeping control was also induced by bad experiences with and a lack of trust in care providers.

The survey showed little interest in self‐management support from primary care nurses and group‐based or online self‐management training courses. Most participants preferred to receive self‐management support from their GP as part of their regular consultations, which is at odds with current policy to give nurses a greater role in supporting self‐management of primary care patients with chronic conditions.^33^, ^34^ In this respect, it is also important to note that contact with social care and community services did not get high priority. Unfamiliarity, patient expectations (expecting medical solutions) and uncertainty about the benefits of these types of care may play a role.^35^ Potential barriers at the patient side need to be carefully addressed when implementing task substitution or integrated care in reforming primary care for people with (multiple) chronic conditions.

Regarding *shared decision making*, participants in the qualitative study mentioned that patients and care providers take other factors into account, or weigh them differently, when taking decisions about treatment and care. This is in line with other studies reporting care preferences and values of (older) people with multimorbidity to differ from those of their care providers.^36^, ^37^, ^38^, ^39^ Defining the value of health services in terms of outcomes that are relevant to patients (in relation to their costs) is the core principle of value‐based health care,^40^ now guiding health‐care transitions worldwide.

The survey showed very few people with multimorbidity wanting to leave decisions about their treatment or care to their GP or other medical specialists. About one third preferred to decide as much as possible themselves and two thirds preferred to take decisions together with their GP or other specialists, reflecting great support for shared decision making. These findings support the results of previous studies that also older people value an active role in decision making about their health and care.^41^

Previous studies have shown *care coordination* to be a core element of high‐quality multimorbidity care,^8^, ^41^, ^42^, ^43^ also highly valued by (older) people with multimorbidity themselves.^20^, ^21^ This was confirmed by our qualitative study revealing that all participants felt care coordination a key element of multimorbidity care. However, not everyone felt a need for a professional coordinator, which was confirmed by the survey showing that the majority of primary care patients with multimorbidity prefer to organize their care and support as much as possible themselves or with their relatives or friends. It seems likely that these people feel capable of coordinating their care, indicating that not all primary care patients with multimorbidity need a professional care coordinator or case manager. Nevertheless, about a third of the participants in the survey preferred a professional to take this role, most often a GP or primary care nurse. These people believed the GP or nurse to have the expertise and also the shortest lines of communication with other care providers, while also having a good overview of their needs.

### 5.2 Strengths and limitations

As in all qualitative studies, we had a limited number of participants in the focus groups and telephone interviews. Their age and gender distributions resembled those of the participants in the survey and of other multimorbid patients registered in Dutch general practices.^44^ However, their self‐rated health was worse (compared with the survey participants), which might explain their motivation to being heard. Moreover, one can expect people willing to participate in focus groups or interviews to be relatively empowered. As such, these people may also be better capable to organize their care.

Qualitative analysis of the focus group and interview data did not reveal elements of care that were felt important by people with multimorbidity that were not covered by the IMCM. However, it is important to note that our focus groups and interviews were guided by this model, which may have narrowed participants’ frame of mind in thinking about important elements of multimorbidity care. Also, the fact that we provided only ten statements to the participants should be taken into account. Their specific formulation may have impacted participants’ weighing of components in determining their priorities. We therefore believe the exact ranking of prioritized components in this study of less importance than the open discussions elicited by the prioritizing exercise, as these provided rich insight into people's underlying values.

The strength of our survey lies in the collection of data from a random selection of patients with multimorbidity receiving care from a number of general practices in the Netherlands that were—at the time of data collection—not involved in an intervention study, as such reflecting the opinions of primary care patients in a real‐world setting. This real‐world setting also brought about some limitations for the study. First, the general practices inviting their multimorbid patients for participation in the survey may not have been representative, considering that these practices had shown an interest in participating in a quality improvement trajectory focusing on multimorbidity care. Furthermore, the real‐world setting also imposed some restrictions to the data collection: not all practices registered the number of excluded patients (and reasons for exclusion) accurately and reminders were not sent to not take up too much of the time of the practice staff. Age and gender distribution of the responders did not point to a substantial violation of representativeness, but self‐rated health seemed on average slightly better than what was found in another study among people (aged 57‐98 years) with three or more chronic conditions in the Netherlands.^45^

### 5.3 Recommendations for future research

As mentioned earlier, values and care preferences of people with multimorbidity are likely to be context‐ and culture‐sensitive.^22^ That is why we could not rely on insights from previous studies conducted in the United States and other non‐European countries. However, this also means that the results we found regarding patients’ care preferences in the context of Dutch primary care cannot be generalized to other settings and countries. We therefore recommend to conduct similar studies in other countries and settings as well. Moreover, we advise local care providers to explore the care preferences of their own patient populations with (multiple) chronic conditions, as practice populations may differ (eg age, ethnicity and socio‐economic situation), which not only impact the population needs, but probably also impact the values and preferences for care. These insights could then be used to develop tailored integrated care at a local scale. Further research of patients’ values and preferences for care may also focus on the role of patients’ health beliefs and perceived health to strengthen the evidence for developing effective care interventions for people with (multiple) chronic conditions.

## 6 CONCLUSION

The JA‐CHRODIS Integrated Multimorbidity Care Model covers elements of care that are considered of great importance by Dutch primary care patients with multimorbidity. As such, the model can be considered suitable to develop high‐quality care for people with multimorbidity in the Netherlands, as has already been done in other European countries. The IMCM shows which elements of care need to be addressed in caring for people with multiple chronic conditions rather than specifying how this should be done, as this will depend on local resources and individual patient preferences.

## CONFLICT OF INTEREST

The authors have no conflict of interest to declare.

## AUTHOR CONTRIBUTIONS

MR designed the study, and was involved in the data collection as a moderator of the two focus groups and as an interviewer; she designed and implemented the patient survey, contributed to the qualitative and quantitative data analysis and wrote the first draft of the paper. RS contributed to the qualitative data analysis and reviewed the draft paper. CL was involved in the data collection as an interviewer, and contributed to writing and reviewing of the draft paper. MJLB was involved in the data collection as a second moderator of one of the focus groups and as an interviewer, and reviewed the draft paper. JK was involved in interpretation of the data and reviewed the draft paper.

## Data Availability

The data that support the findings of this study are available from the corresponding author upon reasonable request.
